# Tanreqing Injection Regulates Cell Function of Hypoxia-Induced Human Pulmonary Artery Smooth Muscle Cells (HPASMCs) through TRPC1/CX3CL1 Signaling Pathway

**DOI:** 10.1155/2022/3235102

**Published:** 2022-02-11

**Authors:** Yonghong Xiang, Fei Zheng, Qinzhe Zhang, RunJuan Zhang, Haiyan Pan, Zongdong Pang, Shimin Dai, Yurong Zhang, Ye Wu, Lunkai Yao, Mengju Su, Luying Lan

**Affiliations:** Affiliated Minzu Hospital of Guangxi Medical University, Department of Pulmonary and Critical Care Medicine, Nanning 530001, China

## Abstract

Hypoxia-induced pulmonary arterial hypertension (HPAH) is due to hypoxia caused by vascular endothelial cell remolding and damage. Previous studies have suggested that CX3CL1 plays an important role in HPAH which is affected by oxidative stress. Ca^2+^ channel activation correlated with increasing NF-*κ*B levels induced by ROS. Tanreqing injection (TRQ) is a traditional Chinese medicine (TCM) for acute upper respiratory tract infection and acute pneumonia. In the present study, we explored the effect of TRQ on human pulmonary artery smooth muscle cells (HPASMCs) undergoing hypoxia and feasible molecular mechanisms involved in. Cell proliferation was assayed using CCK8 kits. Immunofluorescence and western blotting along with ELISA assay were performed to investigate the effect of TRQ on hypoxia-induced ROS, Ca^2+^, hydroxyl free radicals, and the expression of Ca^2+^ channel protein TRPC1, CX3CR1, HIF-1*α*, NF-*κ*Bp65, and p-NF-*κ*Bp65 in HPASMCs. Human CX3CL1 and the inhibitor of TRPC1 as SKF96365 were used for further investigation. TRQ inhibited hypoxia-induced increasing cell adhesion, ROS, Ca^2+^, hydroxyl free radicals, CX3CR1, HIF-1*α*, NF-*κ*Bp65 activation, and even on TRPC1 expression in HPASMC which tended to be attenuated even reversed by CX3CL1. Our results suggested that TRQ might help to attenuate remodeling of HPASMC through inhibiting the ROS and TRPC1/CX3CL1 signaling pathway.

## 1. Introduction

Hypoxia may induce increased pulmonary arterial pressure and pulmonary vascular resistance, which is involved in hypoxia-induced pulmonary arterial hypertension (HPAH) [1, 2]. As for HPAH, there is no specific treatment at present, and heart or lung transplantation may become the only treatment for its late stage, and its mortality and disability rates are high, which has attracted much attention [3, 4]. HPAH is the most important and serious complication of many hypoxic pulmonary diseases, such as chronic obstructive pulmonary disease (COPD), ome (OSAS), and pulmonary fibrosis [5–7]. Complication with HPAH and pulmonary heart disease is one of the most important causes of death [8–10].

HPAH is due to hypoxia-induced vascular endothelial cell damage, pulmonary vasoconstriction (HPV), increased vascular tension, pulmonary vascular remodeling, etc., to result in continuous increasing pulmonary artery pressure and then eventually lead to increasing right ventricular afterload, ventricular remodeling, and heart failure. The pathogenesis of HPAH is very complicated, and there have been many studies on its potential mechanism. At present, it mainly includes the direct factors of hypoxia, humoral factors, ion channels (calcium channels/potassium channels), and gene changes, among which inflammatory reaction, oxidative stress [11, 12], membrane ion channel imbalance [13], and other factors may play a key role. Microthrombosis, the triad of vasoconstriction as well as small pulmonary arteries remodeling, especially vascular remodeling as the most important to drive the pathogenesis of HPAH [14]. The cells in the vasculature layers, particularly as pulmonary artery smooth muscle cells (PASMCs) were concerned, are characterized by hyperproliferation to induced remodeling to lead to increased resistance of blood vessel and result in right heart failure and death eventually [15, 16]. Moreover, abnormal cellular migration of PASMC is closely correlated with vascular remodeling in HPAH [17]. It is therefore essential to find a new way to regulate smooth muscle cell function with uncontrolled proliferation and migration underlying HPH to serve as therapeutic strategies for HPAH.

The transient receptor potential (TRPC) gene family encodes many nonselective cation channels involved in receptor-operated Ca^2+^ channels (ROCC) and store-operated Ca^2+^ channels (SOCC) in vascular smooth muscle cells, which plays pivotal roles in the regulation of PASMC proliferation and migration in HPAH [12, 17]. CX3CL1 and its receptor CX3CRI play an important role in the occurrence and development of HPAH [18, 19], so the study of its signal transduction and regulatory mechanism may provide new clues for the prevention and treatment of HPAH. Results of our previous study indicated that the pulmonary arterial remodeling (PAR) of rats with chronic persistent hypoxia was positively correlated with CX3CL1 in serum and lung tissue, and CX3CL1 was involved in the hypoxic PAR process [20].

Tanreqing injection (TRQ) is a new traditional Chinese medicine (TCM) with good antibacterial, antiviral, antipyretic, and anticonvulsion effects and developed in the treatment of acute upper respiratory tract infection and acute pneumonia. In the present study, we explored the effect of TRQ on human pulmonary Artery Smooth Muscle Cells (HPASMCs) undergoing hypoxia and feasible molecular mechanisms.

## 2. Materials and Methods

### 2.1. Materials

HPASMCs were purchased from ScienCell Research Laboratories, Inc. (USA). DMEM cell culture and fetal bovine serum (FBS) were purchased from Gibco. TRQ (Cat. 1909120) was obtained from Shanghai Kaibao Pharmaceutical Co., Ltd (Shanghai, China). Recombinant Human Fractalkine/CX3CL1 (CX3CL1) and calcium fluorescent probe Fluo-3 AM and N-Acetyl-L-cysteine(cysteine) were purchased from Solarbio (Beijing, China). Reactive oxygen species detection kit and BCA protein concentration assay kit were supplied by Beyotime Co., Ltd. (Shanghai, China). Hydroxyl Free Radical Detection Kit was purchased from Nanjing Jiancheng Bioengineering Institute (Nanjing, China). SKF96365 was purchased from Selleck (Shanghai, China). Antibodies of TRPC1 and CX3CR1 were purchased from Santa Cruz. Antibodies of HIF-1*α*, NF-*κ*Bp65, and p-NF-*κ*Bp65 were purchased from Affinity. Desmin and SMMHC antibodies were purchased from ProteinTech Group, Inc. Human nuclear factor *k*B subunit p65 (NF-*κ*Bp65) ELISA kit was supplied by Jianglai bio. Co. Ltd. Fluor 488-phalloidin reagent was purchased from Abcam (ab176753).

### 2.2. Methods

#### 2.2.1. Cell Culture under Conventional and Hypoxic Conditions and Treatment

HPASMCs were cultured in a medium of DMEM in +10% FBS at 37°C and 5% CO_2_ saturated humidity under regular condition or at 37°C and 2% O_2_ saturated humidity under hypoxia.

Cells were treated with TRQ(0-0.18 crude drug g/ml), SKF96365 (0-500 *μ*M), cysteine (0-100 nM), and/or CX3CL1 (0-600 ng/ml).

#### 2.2.2. MTT Assay

100 *μ*l cell suspension per well with the 4 × 10^3^ cells of the HPASMCs was inserted into a 96-well plate and cultured overnight at 37°C. After the cells adhered to the wall, the original medium was discarded and the cells were treated with TRQ, SKF96365, cysteine, and/or CX3CL1 and cultured in a 37°C and 5% CO_2_ incubator for 24 hours. Then, the treatment medium was discarded and 100 *u*l fresh DMEM medium was added to each well; then, 10 *μ*l MTT was added and cultured at 37°C for 4 h. Suctioning out the medium, adding 150 *μ*l DMSO and shaking for 10 min, the absorbance (OD 568) of each well was determined by the enzyme plate analyzer.

#### 2.2.3. Cell Adhesion

After being treated with TRQ, SKF96365, cysteine, and/or CX3CL1, HPASMCs were collected and the cell density was adjusted to 4 × 10^4^/ml in a serum-free medium. The cells were seeded in a 12-well gummed plate with 1 ml cell suspension per well and cultured in an incubator at 37°C and 5% CO_2_ for 3 hours. After 3 hours, the 12-well plate was removed, and the culture medium was sucked out and washed with PBS for 3 times to remove unadhered cells. Three adherent cells in random view were randomly selected from each well at high power (200x) field to count adherent cells.

#### 2.2.4. Phalloidin Staining

A single layer of cells on slides was stained by Fluor 488-phalloidin reagent (1/1000) for 60 min at room temperature, and nuclei were stained with DAPI. Photographs of cells were taken using a fluorescence photomicrography system (Olympus).

#### 2.2.5. Immunofluorescence

To detect the levels of proteins by immunofluorescence, cells with the single layer on slides were first incubated with primary antibodies against Desmin and SMMHC overnight at 4°C. After then, they were incubated with CY3-labeled sheep against mouse IgG, FITC-labeled sheep, and antirabbit IgG for 1 h at 37°C and washed with PBST three times, each time for 3 min. DAPI was used to counterstain the nucleus. Photographs of cells were taken using a fluorescence photomicrography system (Olympus) and analyzed using ImageJ software.

#### 2.2.6. ROS Assay

ROS of cells was assayed using the Reactive Oxygen Species Assay Kit according to the user's manual. Briefly, the single layer of cells on slides after treatment and/or hypoxia was stained using 10 *μ*M DCFH-DA for 30 min, and then, the photographs of cells were taken using a fluorescence photomicrography system (Olympus).

#### 2.2.7. Hydroxyl Radical Determination

The ability to inhibit hydroxyl radicals of cells was determined using a commercialized kit according to the product's manual. Cell extract was obtained as follows. The adherent cells were gently washed with normal saline and then digested with trypsin. After centrifugation at 1000 × g for 5 min, the cells were collected. The collected cells were washed with cold saline for 3 times. Each group with 1 × 10^6^ cell was resuspended with 200 *μ*l of normal saline, and the cells were then crushed by repeated freeze-thaw process. The extract was centrifuged at 3000 rpm for 10 min, and the supernatant was taken for testing.

To calculate the hydroxyl free radical inhibition ability of the sample, hydroxyl radical inhibition ability (U/Mg protein) = (control OD value − measured OD value)/(standard OD value − blank OD value) × standard concentration (mmol/l) × dilution factor before sample test/(protein concentration of the sample to be tested (Mg protein/ml) × sample volume (0.2 ml)).

#### 2.2.8. Ca Assay

Calcium changes were detected using calcium fluorescent probe Fluo-3 AM. Cells with the single layer on slides were incubated with 0.5 *μ*M Fluo-3 AM solution at 37°C for 60 min, followed by washing three times. After washing, the cells were further incubated for 20 min to ensure complete conversion of Fluo-3AM into Fluo-3. Photographs of cells were taken using a fluorescence photomicrography system (Olympus).

#### 2.2.9. Western Blotting

Cells were collected and lysed with RIPA buffer for protein extraction. After protein quantification using a BCA protein concentration assay kit, equal quantities of proteins were loaded for separation using SDS-PAGE and then transferred to PVDF membranes. Membranes were blocked with 5% defatted milk for 1 h at room temperature and then incubated with primary antibodies (mouse anti-TRCP1 antibody; anti-CX3CR1 antibody; rabbit anti-HIF-1*α* antibody; anti-NF-*κ*Bp65 antibody; and anti-p-NF-*κ*Bp65 antibody) overnight at 4°C. After being washed with TBST, membranes were then incubated with anti-rabbit or anti-mouse horseradish peroxidase- (HRP-) conjugated secondary antibodies for 1 h at room temperature. Blots were detected with an ECL kit (Thermo Fisher Scientific), and images were processed using ImageJ software.

#### 2.2.10. ELISA Assay

The concentration of NF-*κ*Bp65 in the cell supernatant was assayed using the ELISA kit according to the manufacturer. Cell supernatant was collected after centrifugation at 3000 × g for 10 min. 50 *μ*l of each sample was added to the well and incubated at 37°C for 90 min. Add biotinylated antibody working solution 100 *μ*l to each well (prepared within 20 minutes before use), coat the HR-labeled plates with film, and incubate for 60 min at 37°C. Then, the liquid was removed and the plate was washed 3 times, for 30 s each time, about 350 *μ*l/well, dry by spinning, and pat on an absorbent paper to dry the liquid in well. Add 50 *μ*l substrate to each well with film mulching, incubate for 15 min at 37°C, and keep in dark. Add stop solution 50 *μ*l to each well to stop the reaction, and the blue color changes to yellow color. The optical density (OD) value of each hole was measured at 450 nm with a microplate analyzer within 15 minutes. The electric source of the instrument should be opened in advance, the instrument should be preheated, and the detection procedure should be set up.

#### 2.2.11. Statistical Analysis

All data are presented asmean ± SD. The difference between the two groups was analyzed using a no-paired*t*-test, and when among three or more groups, one-way ANOVA analysis was used. All the statistical analyses were performed using GraphPad Prism 8.00 software. When *P* is less than 0.05, the difference was considered significant.

## 3. Results

### 3.1. TRQ, Cysteine, and SKF96365 Inhibited and CX3CX1 Promoted Cell Proliferation of HPASMCs

**Figure 1:**
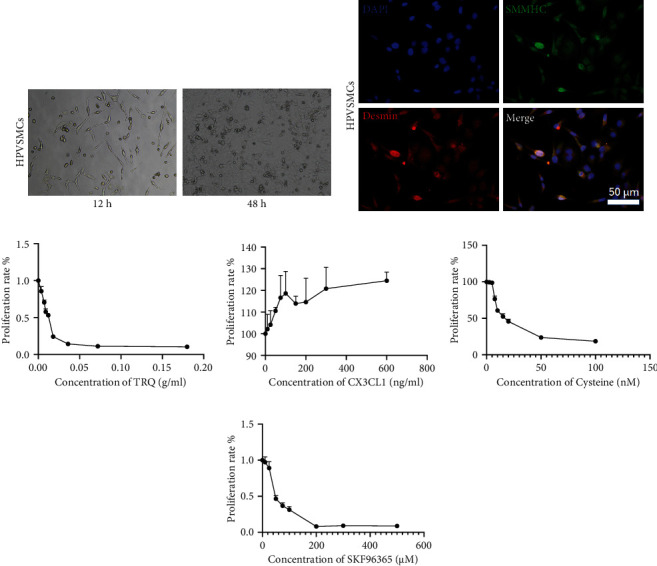
(a) Human pulmonary artery smooth muscle cells (HPASMCs) were cultured for 12 h and 24 h, and the photographs were taken using a light microscope. (b) Immunofluorescence labeling SMMHC (green) and Desmin (red) of HPASMCs. (c) MTT assay was performed to measure cell proliferation of HPASMCs treated with 0-0.18 crude drug g/ml Tanreqing injection (TRQ). (d) MTT assay was performed to measure cell proliferation of HPASMCs treated with CX3CL1 (0-600 ng/ml). (e) MTT assay was performed to measure cell proliferation of HPASMCs treated with cysteine (0-100 nM). (f) MTT assay was performed to measure cell proliferation of HPASMCs treated with SKF96365 (0-500 *μ*M).

Cell proliferation of HPASMCs is involved in pulmonary arterial hypertension, and we explored the effect of TRQ, CX3CX1, cysteine, and TRPC inhibitor SKF96365 on cell proliferation of HPASMCs using MTT assay. At first, immunofluorescence for Desmin and SMMHC protein was performed to confirm HPASMCs (Figures 1(a) and 1(b)). Next, MTT assay was conducted. As shown in Figure 1(c), TRQ inhibited HPASMC cell proliferation in a dose-dependent manner at the concentration of 0-0.18 crude drug g/ml. SKF96365 (0-200 *μ*M) and cysteine (0-100 nM) also showed inhibitive effect on HPASMC cell proliferation dose-dependently (Figures 1(e) and 1(f)). CX3CL1 (0-600 ng/ml) exhibited promoting effect on HPASMC cell proliferation (Figure 1(d)). According to the results of cell proliferation assay, 0.01 crude drug g/ml TRQ, 50 *μ*M SKF96365, 60 nM cysteine, and/or 200 ng/ml CX3CL1 were used in the followed study.

### 3.2. TRQ, Cysteine, and SKF96365 Inhibited and CX3CX1 Promoted Cell Adhesion of HPASMCs under Hypoxia

**Figure 2:**
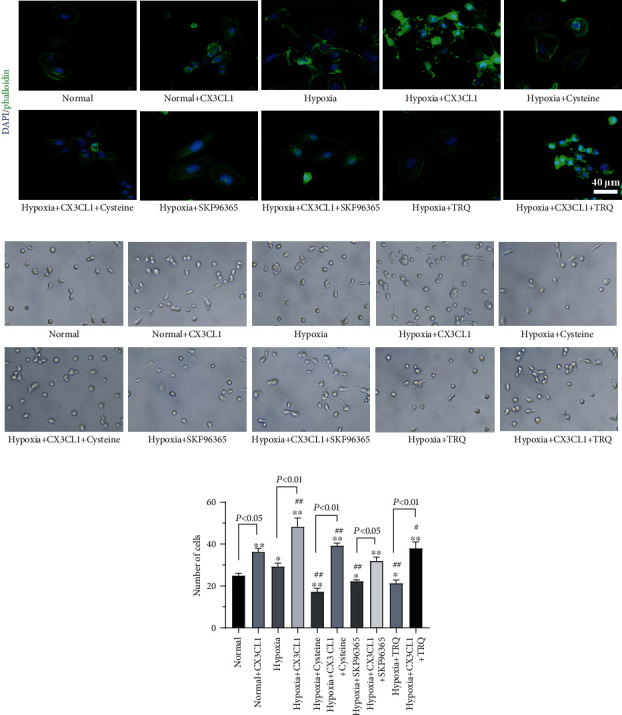
Human pulmonary artery smooth muscle cells (HPASMCs) were treated using 0.01 crude drug g/ml TRQ, 50 *μ*M SKF96365, 60 nM cysteine, and/or 200 ng/ml CX3CL1 and undergoing hypoxia (2% O_2_) for 24 h. (a) Fluorescent-labeled phalloidin was used to label cytoskeleton proteins as F-actin. (b) Cell adhesion assay was performed. (c) Number of adherent cells was analyzed. ^∗^*P* < 0.05 and ^∗∗^*P* < 0.01 vs. normal group. ^#^*P* < 0.05 and ^##^*P* < 0.01 vs. hypoxia group. *n* = 3.

Hypoxia might affect cell adhesion of HPASMCs, which also was correlated with pulmonary arterial hypertension. As well as cell adhesion of HPASMCs being correlated with cell migration, we performed cell adhesion assay in the present study. It was shown in Figure 2(a) fluorescent-labeled phalloidin was used to label cytoskeleton proteins as F-actin, and results indicated that both CX3CL1 treatment and hypoxia-induced cell connections increase the cytoskeleton changing. TRQ, cysteine, and SKF96365 attenuated the induction of either hypoxia or CX3CL1. Cell adhesion assay was similar to TRQ, cysteine, and SKF96365 decreased cell adhesion of HPASMCs induced by hypoxia, and CX3CL1 almost reversed the effect of TRQ, cysteine, or SKF96365 on hypoxia-induced increasing cell adhesion of HPASMCs (Figures 2(b) and 2(c)). In addition, under normal culture conditions, TRQ had no obvious effect on cytoskeleton and cell adhesion of HPASMCs (Supplementary figure 1).

### 3.3. TRQ, Cysteine, and SKF96365 Inhibited and CX3CX1 Promoted Oxidative Stress and Calcium Ion Changes of HPASMCs under Hypoxia

**Figure 3:**
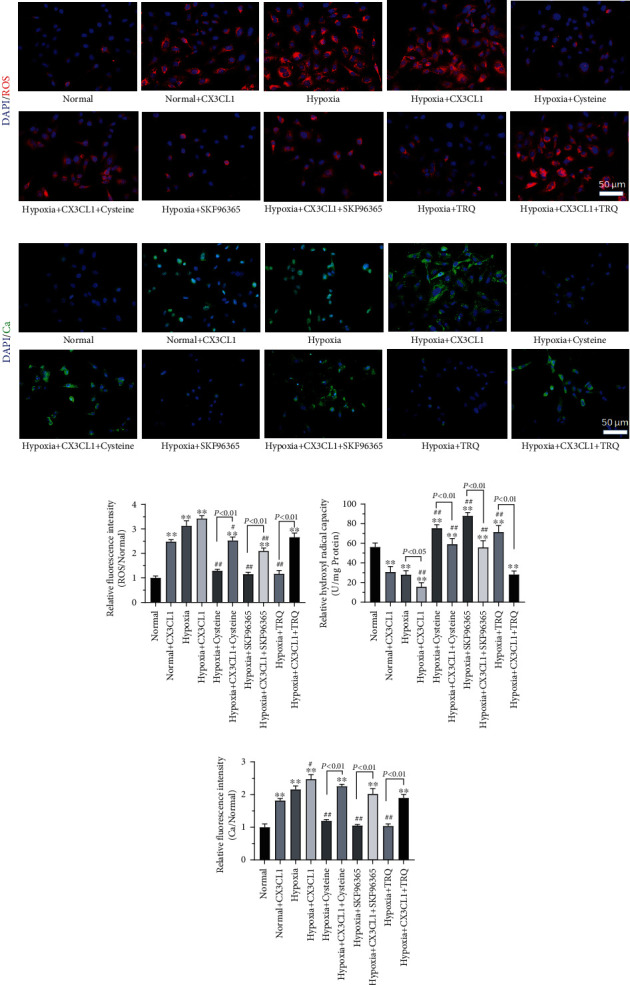
Human pulmonary artery smooth muscle cells (HPASMCs) were treated using 0.01 crude drug g/ml TRQ, 50 *μ*M SKF96365, 60 nM cysteine, and/or 200 ng/ml CX3CL1 and undergoing hypoxia (2% O_2_) for 24 h. (a) ROS of cells was assayed using the Reactive Oxygen Species Assay Kit. Red fluorescence indicated ROS. (b) Calcium (Ca^2+^) changes were detected using calcium fluorescent probe Fluo-3 AM. Green fluorescence indicated Ca^2+^. (c) Relative ROS (to normal group) was analyzed. ^∗^*P* < 0.05 and ^∗∗^*P* < 0.01 vs. normal group. ^#^*P* < 0.05 and ^##^*P* < 0.01 vs. hypoxia group. *n* = 3. (d) The ability to inhibit hydroxyl radicals of cells was determined. ^∗^*P* < 0.05 and ^∗∗^*P* < 0.01 vs. normal group. ^#^*P* < 0.05 and ^##^*P* < 0.01 vs. hypoxia group. *n* = 3. (e) Ca^2+^ was analyzed (to normal group). ^∗^*P* < 0.05 and ^∗∗^*P* < 0.01 vs. normal group. ^#^*P* < 0.05 and ^##^*P* < 0.01 vs. hypoxia group. *n* = 3.

As oxidative stress and calcium ion change as concerned in remolding of HPASMCs in HPAH, ROS, hydroxyl free radicals, and Ca^2+^ changes were explored in our study. Hypoxia induced significantly increasing ROS (Figures 3(a)–3(c)) and Ca^2+^ (Figures 3(b)–3(e)) as well as decreasing the ability to scavenge hydroxyl radicals of HPASMCs (Figure 3(d)). TRQ, cysteine, and SKF96365 inhibited ROS producing and increasing Ca^2+^, while facilitated HPASMC ability to scavenge hydroxyl radical. CX3CL1 may attenuate or even reverse the effects of TRQ, cysteine, or SKF96365 on oxidative stress and calcium ion changes of HPASMCs (*P* < 0.05); furthermore, ROS, hydroxyl free radicals, and Ca^2+^ were not affected by TRQ in normal conditions (Supplementary figure 2).

### 3.4. TRQ Affected Protein Expression of TRPC1, CX3CR1, HIF-1*α*, NF-*κ*Bp65, and p-NF-*κ*Bp65 under Hypoxia

**Figure 4:**
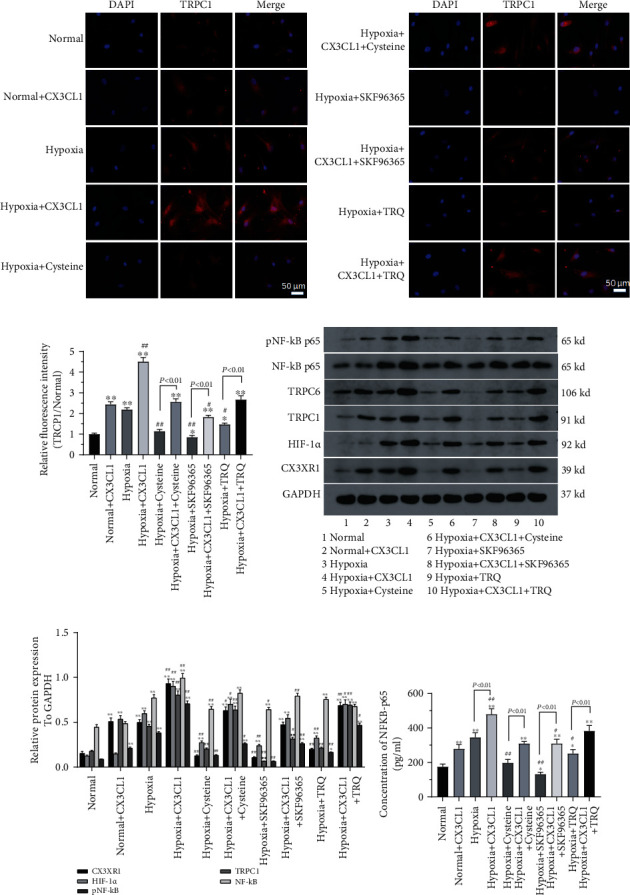
Human pulmonary artery smooth muscle cells (HPASMCs) were treated using 0.01 crude drug g/ml TRQ, 50 *μ*M SKF96365, 60 nM cysteine, and/or 200 ng/ml CX3CL1 and undergoing hypoxia (2% O_2_) for 24 h. (a) Protein expression of TRPC1 was detected by immunofluorescence. (b) Relative TRPC1 was analyzed (to normal) ^∗^*P* < 0.05 and ^∗∗^*P* < 0.01 vs. normal group. ^#^*P* < 0.05 and ^##^*P* < 0.01 vs. hypoxia group. *n* = 3. (c) The expression of TRCP1, CX3CR1, HIF-1*α*, NF-*κ*Bp65, and p-NF-*κ*Bp65 was measured using western blotting. (d) The expression of TRCP1, CX3CR1, HIF-1*α*, NF-*κ*Bp65, and p-NF-*κ*Bp65 was analyzed from blots. ^∗^*P* < 0.05 and ^∗∗^*P* < 0.01 vs. normal group. ^#^*P* < 0.05 and ^##^*P* < 0.01 vs. hypoxia group. *n* = 3. (e) The level of NF-*κ*Bp65 in cell supernatant was assayed using the ELISA kit. ^∗^*P* < 0.05 and ^∗∗^*P* < 0.01 vs. normal group. ^#^*P* < 0.05 and ^##^*P* < 0.01 vs. hypoxia group. *n* = 3.

The possible molecular pathways involved in the TRQ role in HPASMCs under hypoxia were also explored in the present study. Protein expression of TRPC1, CX3CR1, HIF-1*α*, NF-*κ*Bp65, and p-NF-*κ*Bp65 was measured using immunofluorescence assay, western blotting, and ELISA assay. Results showed that hypoxia induced TRPC1, CX3CR1, HIF-1*α*, NF-*κ*Bp65, and p-NF-*κ*Bp65 upregulating in HPASMCs (Figures 4(a)–4(d)). TRQ, cysteine, or SKF96365 attenuated upregulation of TRCP1, CX3CR1, HIF-1*α*, and p-NF-*κ*Bp65 expression significantly (*P* < 0.05), all of which tended to be reversed by CX3CL1 in HPASMCs under hypoxia. It was noted that there was no significant change in protein expression of NF-*κ*Bp65 in HPASMCs, but significant difference was found in cell supernatant of HPASMCs among all the treated groups (Figure 4(e)); simultaneously, immunofluorescent staining of TPPC1 in normal conditions treated with TRQ was also carried out. Results showed that fluorescence intensity did not change significantly by TRQ (Supplementary figure 3).

## 4. Discussion

HPAH is a group of pathophysiological syndromes characterized by increased pulmonary arterial pressure and pulmonary vascular resistance caused by hypoxia. Direct factors of hypoxia, oxidative stress, and ion channels (calcium channels/potassium channels) play key roles in HPAH [11, 21]. As one of the main pathological changes of HPAH, PAR was correlated with migration and proliferation of pulmonary artery smooth muscle cells (PASMCs) [21]. Multiple previous studies have confirmed the involvement of oxidative stress in PAR and HPAH [21–23]. In the present study, hypoxia induced HPASMCs to simulate the environment of PASMCs in HPAH.

Previous studies have suggested that CX3CL1 plays an important role in hypoxic PAR [24, 25]. CX3CL1 acts as both chemokine and intercellular adhesion molecule and plays a role in a variety of diseases due to its complex biological characteristics. CX3CL1 is involved in various pathological processes, such as inflammatory response [26, 27], and is closely related to the occurrence and development of such pulmonary diseases. CX3CL1 and its receptor CX3CRI play an important role in the occurrence and development of HPAH [18, 19], so the study of its signal transduction and regulatory mechanism may provide new clues for the prevention and treatment of HPAH. It was found that higher levels of CX3CL1-CX3CR1 and plasma soluble CX3CL1 in patients with pulmonary hypertension than in the control group and the expression and function of CX3CL1 in circulating T cells were upregulated [28]. Long-term chronic hypoxia can induce upregulation of CX3CL1 mRNA expression in lung tissues and pulmonary arteries and increase the synthesis and secretion of CX3CR1 protein. CX3CR1 and TNF-*α* are also highly expressed in patients with chronic pulmonale (Zhang et al.). In our previous study [20], it was found that the PAR of rats with chronic persistent hypoxia (CH) was positively correlated with CX3CL1 in serum and lung tissue, and CX3CL1 was involved in the hypoxic PAR process. It has been confirmed in a number of studies that oxidative stress participates in PAR and HPAH [15, 23, 29]. Further previous studies [30] showed that serum CX3CL1 level in patients with chronic obstructive pulmonary disease (COPD) was positively correlated with nuclear factor-*k*B (NF-*κ*B), ox-LDL, and pulmonary arterial pressure and negatively correlated with SOD, which indicated that CX3CL1 was closely correlated with oxidative stress. CX3CL1 may serve as one of new treatment strategies for HPAH. Our results from the present study indicated that hypoxia induced increasing ROS and hydroxyl free radicals as well as the activity of NF-*κ*B (p-NF-*κ*Bp65) and CX3CR1 protein in HPASMCs which may be attenuated by TRQ and cysteine.

It is suggested that Ca^2+^ channel activation is correlated with increasing NF-*κ*B and TNF-*α* levels induced by ROS [31]. Ca^2+^ channels are key initiators of cell proliferation-related signal transduction. Calcium signal transduction plays a particularly important role in pulmonary vasoconstriction. A large number of studies have found that ion balance in PASMC, especially changes in calcium metabolism, is an important factor in the regulation of various pathophysiological changes in hypoxic pulmonary hypertension [32, 33]. It has been confirmed that both PAR and HPAH induced by hypoxia are closely related to Ca^2+^-mediated membrane channels [32, 34, 35]. The decreasing oxygen concentration can enhance the expression of Ca^2+^ channel in pulmonary artery resistance vessels and increase the intracellular Ca^2+^ concentration, which causes the imbalance of cell membrane homeostasis, thus activating various nuclear transcription factors, promoting the contraction and proliferation of PASMC, and finally leading to PAR and HPAH [34, 35]. The effect of TRQ on Ca^2+^ changes in HPASMCs under hypoxia was therefore explored in our study. It was similar to ROS; hypoxia induced increasing Ca^2+^ which was inhibited by TRQ and cysteine in HPASMCs. CX3CL1 exhibited attenuation to the effect of TRQ and cysteine on Ca^2+^ induced by hypoxia.

TRP is a new type of ion channel discovered in recent years, which plays an important role in cardiometabolic diseases. There are about ten TRP channels (TRPC) with different functions distributed in vascular smooth muscle cells [36]. TRP channels can be activated by various physical and chemical stimuli, such as ROS, calcium pool-dependent calcium ion influx (SOCE), receptor-dependent calcium ion influx (ROCE), and mechanical forces. TRPC consists of seven members: TRPC1-7. The expression of TRPC1 and TRPC6 is most abundant in the smooth muscle of rat, mouse, and human pulmonary artery, and TRPCI and TRPC6 are closely related to hypoxic pulmonary hypertension. TRPC1 is closely related to HPH, hypertension, and tumor [37, 38]. It has been found that TRPC1 is expressed in mouse PASMC, and after 3 weeks of hypoxia, the pulmonary artery of mice is remodeled with changed function, and the protein level of TRPCI is upregulated in PASMC [39]. Involvement of TRPC1 in mediating SOCE and ROCE and the upregulation of pulmonary artery basal tension were confirmed on PASMC by RNA interference and nonspecific TRP channel inhibitors [40]. In addition, TRPC-dependent calcium influx may also be involved in various types of pulmonary hypertension [41, 42]. We then explored the effect of TRQ on TRPC1 expression in hypoxia-induced HPASMC, and the inhibitor of TRPC1 as SKF96365 was also used for further investigation. Our results indicated that the effect of TRQ was analogous to SKF96365 on hypoxia-induced ROS, Ca^2+^, hydroxyl free radicals, CX3CR1, HIF-1*α*, NF-*κ*Bp65, p-NF-*κ*Bp65, and even on TRPC1 expression in HPASMC.

In conclusion, TRQ inhibited hypoxia-induced increasing cell adhesion, ROS, Ca^2+^, hydroxyl free radicals, CX3CR1, HIF-1*α*, NF-*κ*Bp65 activation, and even on TRPC1 expression in HPASMC, which tend to be attenuated even reversed by CX3CL1. Our results suggested that TRQ might help to attenuate remodeling of HPASMC through inhibiting ROS and TRPC1/CX3CL1 signaling pathway involved in. However, this study has many limitations as *in vitro* cell experiments cannot completely simulate pathological conditions *in vivo*. Therefore, the effect of TRQ on chronic hypoxic pulmonary hypertension in rat model would be explored in our further study, results of which will provide basis for clinical experiment.

## Data Availability

The data in this study can be obtained from the corresponding author.

## Supplementary Materials

Supplementary Materials Figure S1: human pulmonary artery smooth muscle cells (HPASMCs) were treated using 0.01 crude drug g/ml TRQ and were undergoing normoxia or hypoxia (2% O_2_) for 24 h. Fluorescent-labeled phalloidin to label cytoskeleton proteins as F-actin and cell adhesion. Figure S2: ROS, calcium (Ca_2+_), and the ability to inhibit hydroxyl radicals of cells. Figure S3: protein expression of TRPC1 detected by immunofluorescence.
