# Supplementary material for: Tanreqing Injection Regulates Cell Function of Hypoxia-Induced Human Pulmonary Artery Smooth Muscle Cells (HPASMCs) through TRPC1/CX3CL1 Signaling Pathway
Source: Oxid Med Cell Longev. 2022 Feb 11;2022:3235102. doi: 10.1155/2022/3235102 (PMC8856792; doi:10.1155/2022/3235102)
Supplement: Supplementary Materials — Figure S1: human pulmonary artery smooth muscle cells (HPASMCs) were treated using 0.01 crude drug g/ml TRQ and were undergoing normoxia or hypoxia (2% O2) for 24 h. Fluorescent-labeled phalloidin to label cytoskeleton proteins as F-actin and cell adhesion. Figure S2: ROS, calcium (Ca2+), and the ability to inhibit hydroxyl radicals of cells. Figure S3: protein expression of TRPC1 detected by immunofluorescence. [file 3235102.f1.docx]

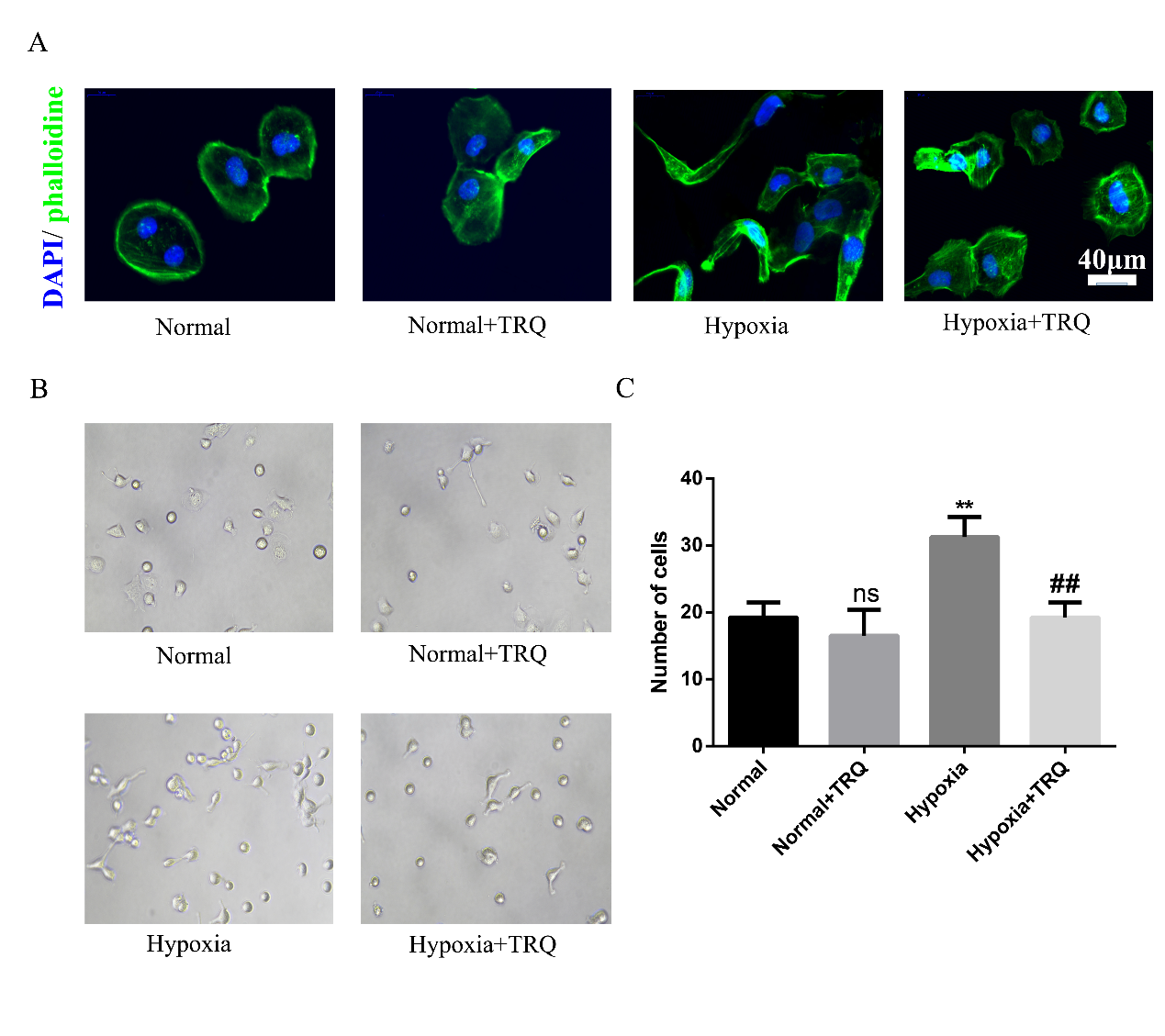


**Supplementary figure1**

Human pulmonary Artery Smooth Muscle Cells (HPASMCs) were treated using 0.01 crude drug g/ml TRQ and undergoing normoxia or hypoxia (2% O_2_) for 24h. **(A)** Fluorescent labeled phalloidine was used to label cytoskeleton proteins as F-actin. (B-C) Cell adhesion assay was performed, and number of adherent cells were analyzed. ***P*＜0.01 vs Normal group, ##*P*＜0.01 vs Hypoxia group. n=3.


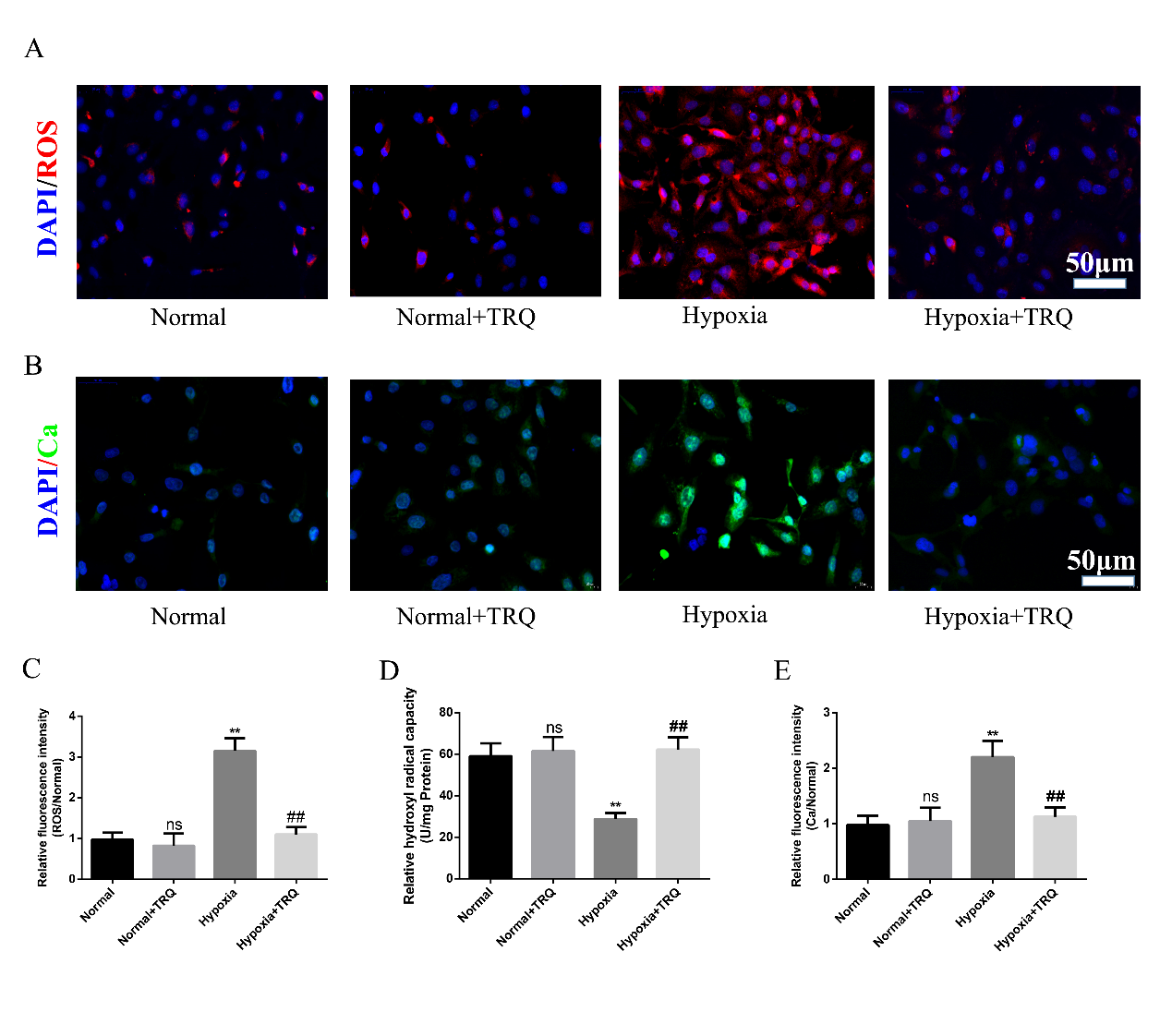


**Supplementary figure2**

Human pulmonary Artery Smooth Muscle Cells (HPASMCs) were treated using 0.01 crude drug g/ml TRQ and undergoing normoxia or hypoxia (2% O_2_) for 24h. (A) Ros of cells were assayed using Reactive Oxygen Species Assay Kit. Red fluorescence indicated ROS. (B) Calcium (Ca2+) changes were detected using calcium fluorescent probe Fluo-3AM. Green fluorescence indicated Ca2+. (C) Relative ROS (To normal group) was analyzed. (D) The ability to inhibit hydroxyl radicals of cells was determined. (E) Ca2+ was analyzed (To normal group). ***P*＜0.01 vs Normal group. ## *P*＜0.01 vs Hypoxia group. n=3.


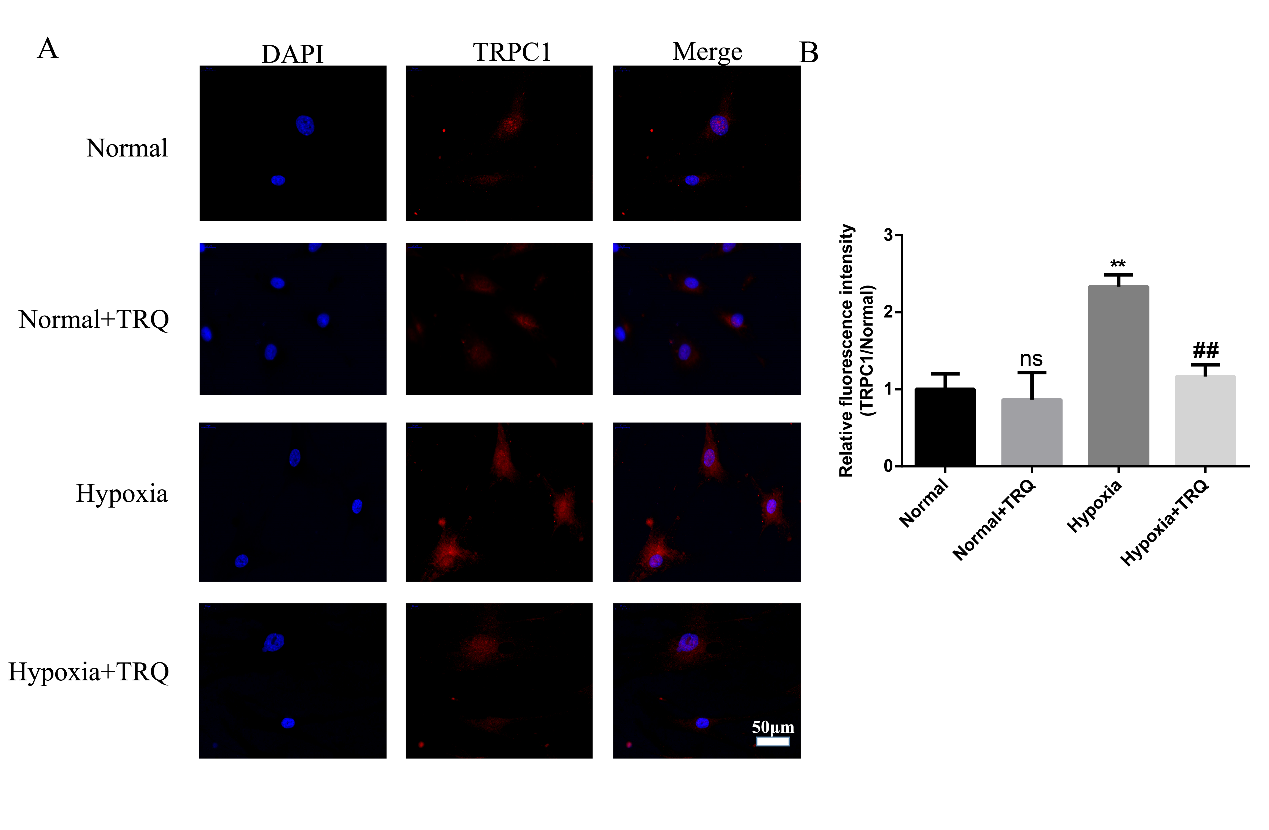


**Supplementary figure3**

Human pulmonary Artery Smooth Muscle Cells (HPASMCs) were treated using 0.01 crude drug g/ml TRQ and undergoing normoxia or hypoxia (2% O_2)_ for 24h. (A) Protein expression of TRPC1 was detected by immunofluorescence. (B) Quantitative analysis of fluorescence intensity, ***P*＜0.01 vs Normal group. ## *P*＜0.01 vs Hypoxia group. n=3.
